# High Epoxidation Yields of Vegetable Oil Hydrolyzates and Methyl Esters by Selected Fungal Peroxygenases

**DOI:** 10.3389/fbioe.2020.605854

**Published:** 2021-01-05

**Authors:** Alejandro González-Benjumea, Gisela Marques, Owik M. Herold-Majumdar, Jan Kiebist, Katrin Scheibner, José C. del Río, Angel T. Martínez, Ana Gutiérrez

**Affiliations:** ^1^Instituto de Recursos Naturales y Agrobiología de Sevilla (IRNAS), CSIC, Seville, Spain; ^2^Novozymes A/S, Bagsvaerd, Denmark; ^3^JenaBios GmbH, Jena, Germany; ^4^Centro de Investigaciones Biológicas Margarita Salas (CIB), CSIC, Madrid, Spain

**Keywords:** epoxidation, vegetable oils, enzymes, peroxygenases, polyunsaturated fatty acids, fatty acid methyl esters, complex lipid mixtures, biocatalysis

## Abstract

Epoxides of vegetable oils and free and methylated fatty acids are of interest for several industrial applications. In the present work, refined rapeseed, sunflower, soybean, and linseed oils, with very different profiles of mono- and poly-unsaturated fatty acids, were saponified and transesterified, and the products treated with wild unspecific peroxygenases (UPOs, EC 1.11.2.1) from the ascomycete *Chaetomium globosum* (*Cgl*UPO) and the basidiomycete *Marasmius rotula* (*Mro*UPO), as well as with recombinant UPO of the ascomycete *Humicola insolens* (r*Hin*UPO), as an alternative to chemical epoxidation that is non-selective and requires strongly acidic conditions. The three enzymes were able of converting the free fatty acids and the methyl esters from the oils into epoxide derivatives, although significant differences in the oxygenation selectivities were observed between them. While *Cgl*UPO selectively produced “pure” epoxides (monoepoxides and/or diepoxides), *Mro*UPO formed also hydroxylated derivatives of these epoxides, especially in the case of the oil hydrolyzates. Hydroxylated derivatives of non-epoxidized unsaturated fatty acids were practically absent in all cases, due to the preference of the three UPOs selected for this study to form the epoxides. Moreover, r*Hin*UPO, in addition to forming monoepoxides and diepoxides of oleic and linoleic acid (and their methyl esters), respectively, like the other two UPOs, was capable of yielding the triepoxides of α-linolenic acid and its methyl ester. These enzymes appear as promising biocatalysts for the environmentally friendly production of reactive fatty-acid epoxides given their self-sufficient monooxygenase activity with selectivity toward epoxidation, and the ability to epoxidize, not only isolated pure fatty acids, but also complex mixtures from oil hydrolysis or transesterification containing different combinations of unsaturated (and saturated) fatty acids.

## Introduction

In the search for sustainable chemistry, considerable attention is being paid to renewable feedstocks and, among these, vegetable oils represent a great proportion of the current consumption by the chemical industry. However, the chemical possibilities of renewable oils and fats are still very far from being fully exploited. Indeed, most of oleochemical reactions have been those occurring at the fatty acid carboxy group, while only a very minor proportion of them have involved transformations of the alkyl chain. Nevertheless, the latter reactions have great potential for extending the range of compounds available from oils and fats.

In this context, the epoxidation of vegetable oils is being studied due to the current and potential commercial applications of the products obtained. Oil epoxides are used as stabilizers and plasticizers (Kandula et al., 2014; Jia et al., 2016), and are also promising intermediates for the production of polyols (Zhang et al., 2014a), polyurethanes (Zhang et al., 2014b), biolubricants (Borugadda and Goud, 2014), and epoxy resins (Xia and Larock, 2010), among other uses. Moreover, by simple industrial procedures, fatty acids are available from vegetable oils in such purity that they may be used for further chemical transformations. Their conversion to fatty-acid methyl esters (FAMEs) is a well-known application, largely investigated for biodiesel production. Moreover, unsaturated fatty acids and FAMEs can be further epoxidized, and used in industrial syntheses of chemicals and intermediates.

Industrial epoxidation of unsaturated fatty-acid compounds is generally performed by the Prileschajew (1909) reaction via percarboxylic acids. However, this method, which includes strong mineral acids as catalysts for the “*in situ*” generation of peracids, suffers from several drawbacks such as the low selectivity for epoxide formation, due to the oxirane ring opening in acidic medium, the corrosive nature of acids, and the unstable character of peracids (Danov et al., 2017). New methods have been investigated aimed at searching an alternative, such as the chemo-enzymatic synthesis with lipases catalyzing carboxylic acid reaction with H_2_O_2_ yielding the reactive peracids (Björkling et al., 1992; Tiran et al., 2008). On the other hand, direct enzymatic processes emerge as a solution for more selective and environmentally friendly epoxidation of unsaturated lipids. Several enzymes are known to catalyze epoxidation directly, such as some cytochrome P450 monooxygenases (P450), diiron-center oxygenases, and plant peroxygenases (Ruettinger and Fulco, 1981; Oliw, 1994; Piazza et al., 2003). However, they often present some drawbacks, such as their intracellular nature and frequent association to membranes (in the case of plant peroxygenases and some P450s), and the requirement for costly cosubstrates or FAD-containing auxiliary enzymes or domains (in the case of P450s and diiron oxygenases).

Phylogenetically distant fungal unspecific peroxygenases (UPOs, EC 1.11.2.1) are related to P450s in the sense that they also contain a heme prosthetic group coordinated by a cysteine ligand, but they do not depend on the reductive activation of molecular oxygen but catalyze the transfer of an oxygen atom from peroxides to reducing substrates (Hofrichter et al., 2015). The first UPO was described in the basidiomycete *Agrocybe aegerita* (*Aae*UPO) (Ullrich et al., 2004) and since then, UPO enzymes have been purified from other Basidiomycota and Ascomycota species such as *Coprinellus radians* (*Cra*UPO) (Anh et al., 2007), *Marasmius rotula* (*Mro*UPO) (Gröbe et al., 2011), and *Chaetomium globosum* (*Cgl*UPO) (Kiebist et al., 2017), which is indicative for their widespread occurrence in the fungal kingdom. In addition to these wild (i.e., non-recombinant) enzymes, there are other UPOs, e.g., from *Coprinopsis cinerea* (r*Cci*UPO) (Babot et al., 2013) and *Humicola insolens* (r*Hin*UPO) (Kiebist et al., 2017), that are only known as recombinant proteins heterologously expressed by Novozymes A/S (Bagsvaerd, Denmark) in the mold *Aspergillus oryzae* (Landvick et al., 2016), and very recently additional UPOs heterologously expressed in *Escherichia coli* (Linde et al., 2020). Initially, UPO enzymes were shown to catalyze oxygenation reactions on aromatic compounds (Hofrichter et al., 2010), and their action on aliphatic compounds was demonstrated later (Gutiérrez et al., 2011; Peter et al., 2011).

Here, we demonstrate a promising enzymatic technology to epoxidize, under mild and environmentally friendly conditions, complex mixtures of free and methylated fatty acids from representative vegetable oils, which had been previously applied on isolated pure fatty acids (Aranda et al., 2018), for its industrial application in the production of biobased binder ingredients, in collaboration with interested companies. This includes the use of two wild UPOs, namely *Mro*UPO and *Cgl*UPO, and recombinant r*Hin*UPO, all of them with preferential epoxidation (vs. hydroxylation) oxygenation patterns. These and related fungal peroxygenases elude some of the limitations of other monooxygenases since they are secreted proteins, therefore far more stable, and only require H_2_O_2_ for activation (Wang et al., 2017; Hofrichter et al., 2020). Moreover, their recent expression as soluble and active enzymes in *Escherichia coli* is expanding the number of UPO enzymes available from related genes in sequenced genomes (Linde et al., 2020) and, simultaneously, enabling the rational design of the available UPOs as *ad hoc* biocatalysts of industrial interest, using the protein engineering tools (Carro et al., 2019; González-Benjumea et al., 2020; Municoy et al., 2020).

## Materials and Methods

### Oils

Four refined vegetable oils—namely rapeseed, soybean, sunflower, and linseed oils—were provided by the Cargill company and stored at 4°C, before their saponification, transesterification and use as UPO substrates. For characterization of the whole lipid profiles (“intact” lipids), aliquots were directly treated with BSTFA [*N*,*O*-bis-(trimethylsilyl)trifluoroacetamide] at 80°C for 1 h, and analyzed by GC-MS.

### Enzymes

*Mro*UPO and *Cgl*UPO are wild enzymes isolated at JenaBios (Jena, Germany) from pure cultures of *M. rotula* DSM 25031 and *C. globosum* DSM 62110 from the German Collection of Microorganisms and Cell Cultures (Braunschweig, Germany). r*Hin*UPO is a recombinant enzyme obtained at Novozymes A/S (Bagsvaerd, Denmark) (Kiebist et al., 2017), by heterologous expression of the cloned gene in the *A. oryzae* industrial host, using proprietary technology (Landvick et al., 2016). In all cases, the secreted enzyme was recovered after eliminating the fungal mycelium by filtration of liquid cultures, concentrated by ultrafiltration or ammonium sulfate precipitation, and purified by successive steps of fast protein liquid chromatography (FPLC) using Äkta systems (GE Healthcare) and different ion-exchange and size-exclusion columns till apparent homogeneity. This was confirmed by sodium dodecylsulfate-polyacrylamide gel electrophoresis under denaturing conditions, and presence of the Soret band characteristic of heme-thiolate proteins at 418 nm of their UV-visible spectra. In the case of *Mro*UPO, Q-Sepharose FF, Source 15Q, and Superdex-75 columns were used, and the purified enzyme presented a molecular mass of 32 kDa (Gröbe et al., 2011). Purified *Cgl*UPO showed a molecular mass of 36 kDa (Kiebist et al., 2017), while r*Hin*UPO presents a theoretical molecular mass of 29 kDa according to its reported amino-acid sequence (Lund et al., 2013). In all cases, the enzyme concentrations were estimated from the characteristic spectrum of peroxygenase complex with carbon monoxide (Otey, 2003).

### Chemicals

All chemicals and substrates were purchased from Merck except the following *cis*-epoxide standards: 9,10-epoxyoctadecanoic acid and its methyl ester and 9,10-epoxyoctadec-12(*Z*)-enoic acid from Santa Cruz Biotechnology; 12,13-epoxyoctadec-9(*Z*)-enoic acid from Cayman; and 9,10–12,13-diepoxyoctadecanoic acid from Larodan.

Mono, di, and triepoxides from α-linolenic acid were synthesized with *meta*-chloroperbenzoic acid (in 1.3, 2.7, and 4.5 molar excess for mono-, di-, and tri-epoxides, respectively), in CHCl_3_ at room temperature for 1 h. The reaction was quenched with saturated NaHCO_3_ and the organic layer was recovered and dried with MgSO_4_ before GC-MS analysis. Methyl esters of the above epoxy-fatty acids were synthesized using trimethylsilyldiazomethane (2 equiv) in MeOH-ether (2:3) at room temperature for 30 min. The solution was dried under N_2_ and dissolved in CHCl_3_ for GC-MS analysis.

### Hydrolysis and Transesterification of Vegetable Oils

For achieving an accurate quantification of triglyceride fatty acids in the vegetable oils, saponification, and transesterification reactions were performed as described below, and the resulting free fatty-acid and FAMEs were analyzed by GC-MS.

Saponification was carried out by preparing a suspension of the vegetable oils (100 mg) in a freshly prepared solution (1.5 mL) of 0.5 M KOH in absolute EtOH, and refluxing for 30 min. The solution was acidified to pH 3 with HCl, and extracted three times with *n*-hexane in a separation funnel. The mixture was stored at 4°C, and trimethylsilyl (TMS) derivatives were prepared by BSTFA treatment before analysis by GC-MS.

Lipid conversion into FAMEs was done by preparing a suspension of the vegetable oils (100 mg) in a freshly prepared solution (4 mL) of 0.5 M NaOMe in anhydrous MeOH, and heating to 50°C for 30 min, with vigorous shaking (each 5 min). Then, the reaction was quenched with glacial AcOH (200 μL) and diluted with H_2_O (8 mL). Finally, FAMEs were extracted three times with *n*-hexane. The mixture was stored at 4°C, and analyzed without further derivatization. From the FAME yields obtained, and average purity (triglyceride content) of the different vegetable oils analyzed near 99% could be estimated.

### Enzymatic Reactions

For UPO reactions (1 mL) with saponified oils (0.1 mM), the saponified sample (0.1 μmol) was solved in acetone and diluted with sodium phosphate buffer, pH 5.5 (*Mro*UPO) or 7.0 (*Cgl*UPO and r*Hin*UPO). After addition of the enzyme (0.1 nmol) the solution was heated to 30°C, and the reaction was triggered by adding aqueous H_2_O_2_ (1.25 μmol) in pulses for 30 min. Taking advantage from previous studies on fatty-acid oxygenation by UPOs (Gutiérrez et al., 2011; Babot et al., 2013; Aranda et al., 2018; Carro et al., 2019; González-Benjumea et al., 2020; Municoy et al., 2020), acetone at a concentration of 20% (v/v) was used as cosolvent. The reactions with transesterified oils were carried out following a similar procedure for 2 h. The enzyme (0.5 or 1 nmol) was added in a split dose (at the beginning and after 1 h) to maximize the conversion, and the solution was heated to 40°C. H_2_O_2_ (1.25–5 μmol) was added in pulses, although a syringe pump was also tested. The acetone concentration was 40% (v/v). Control experiments in which saponified and transesterified oil samples were treated under the same conditions (including H_2_O_2_), but without enzyme, were also performed. In all cases, the products were extracted with methyl *tert*-butyl ether (MTBE) and dried under N_2_. BSTFA was used to prepare TMS derivatives that were analyzed by GC-MS.

In scaling-up experiments of enzymatic epoxidation of saponified sunflower oil, the substrate concentration could be increased up to 30 mM (buffer pH 7.0 and 40% acetone) and the enzyme dose was 30 μM, which means the same substrate/enzyme ratio previously used. The concentration of H_2_O_2_ was 234.0 mM (5.5 equiv) for *Cgl*UPO and 93.5 mM (2.1 equiv) for *Mro*UPO and r*Hin*UPO. In all cases, the oxidant was slowly added with a syringe pump and the reaction was heated to 30°C. The reaction time was 2.5 h with *Mro*UPO and 1 h with *Cgl*UPO and r*Hin*UPO. The products were recovered with MTBE and dried in a rotary evaporator. Reaction volumes up to 100 mL were tested with *Mro*UPO. Because of the optimum pH for *Mro*UPO, the scale-up was also performed at pH 5.5. In this case, the maximal substrate loading was 4 mM (55% acetone), the enzyme dose was 4 μM and the oxidant was 12.5 mM (2.1 equiv) with identical reaction time.

All the enzymatic reactions were performed in duplicate, or triplicate if required, and the dispersion of the results after the GC-MS analysis described below, was always below 10% of the corresponding mean values. Fatty-acid conversions were calculated by comparing the remaining (unreacted) substrate in the UPO reactions with the same substrate in the control experiments. Epoxidation yields were calculated taking into account the products obtained for each substrate and their epoxidation degree. Then, the result were corrected according to the conversion (see above) calculated for each substrate. Selectivities (between family products or diastereomers) were calculated taking into account the product/s of interest, with respect to all the products formed from one initial substrate.

### GC-MS Analyses

The GC-MS analyses of “intact” lipids were performed on a Star 3400 gas chromatograph (Varian, Walnut Creek, CA, United States) equipped with a capillary column (DB-5HT, 12 m × 0.25 mm internal diameter, 0.1 μm film thickness; J&W Scientific, Santa Clara, CA, United States), and coupled with an ion-trap detector (Varian Saturn 400). Helium was used as carrier gas at a rate of 2.0 mL min^–1^. The injector was set to 120°C during the injection and, 0.1 min after it was programmed to 380°C at a rate of 200°C min^–1^ and held for 10 min. The oven was heated from 120°C (1 min) to 380°C (5 min) at 10°C min^–1^, and the transfer line was set to 300°C. Compounds were identified by mass fragmentography and by mass spectra comparison with those of the Wiley and NIST libraries, and with authentic standards. Quantification was accomplished from total-ion (or characteristic-ion) peak areas, using molar response factors of the same or similar compounds. For quantification of triglyceride conversion, trilinolein was used in a concentration range between 0.01 and 0.8 mg⋅mL^–1^ to elaborate calibration curves.

The GC-MS analyses of fatty acids were performed with a Shimadzu GC-MS QP2010 Ultra equipment, using the same capillary column mentioned above but with longer length (30 m). Helium was used at a rate of 0.83 mL min^–1^. The injection was performed at 300°C, the oven was heated from 120°C (1 min) to 300°C (15 min) at 5°C min^–1^, and the transfer line was kept at 300°C. Compounds were identified and quantified as described above. Methyl palmitate, methyl stearate, methyl oleate, methyl linoleate, and methyl α-linolenate were used in a concentration range between 0.004 and 0.05 mg mL^–1^ to obtain calibration curves.

## Results and Discussion

### Chemical Characterization of Whole Oils, Oil Hydrolyzates, and Methyl Esters

Samples of rapeseed, soybean, sunflower, and linseed vegetable oils provided by Cargill were first analyzed by GC-MS as whole oils (i.e., without prior hydrolysis or transesterification). As shown under the chromatographic conditions used (12 m columns and high temperature programs), the refined oils consisted exclusively of triglycerides. The composition of each of them, determined by mass fragmentography using characteristic single ions (profiles not shown), revealed the presence of triglycerides containing one (T1), two (T2), and three unsaturated (T3) fatty acids (Supplementary Figure S1). However, quantification of the different triglycerides that coelute in the same peak is not possible under these conditions.

With the aim of achieving an accurate quantification of fatty acids in triglycerides, saponification and transesterification of vegetable oils were performed, and the fatty-acid and FAME patterns were analyzed by GC-MS. To ensure that oils were completely hydrolyzed/transesterified, all reactions were first analyzed under the chromatographic conditions for analysis of “intact” lipids (using 12 m short columns) (Supplementary Figure S2). After checking complete reactions, quantification of individual acids and esters was performed under the corresponding chromatographic conditions (using 30 m columns).

The GC-MS analyses (Table 1) revealed that all oils analyzed are composed of C16 and C18 fatty acids—namely palmitic, stearic, oleic, linoleic, and α-linolenic acids—and present high purities (estimated triglyceride content near 99%) as expected for refined oils. Soybean oil has the highest abundance of saturated fatty acids and a good concentration of polyunsaturated fatty acids (PUFAs), almost exclusively linoleic acid. In contrast, rapeseed oil has a relatively low content of saturated fatty acids but also a low content on PUFAs, due to the high proportion of oleic acid. On the other hand, sunflower and linseed oils combine a moderated or low profile of saturated fatty acids and a high concentration in PUFAs. In principle, this makes them good candidates for epoxidation reactions, especially linseed oil, with high content of triunsaturated α-linolenic acid. The samples containing free fatty acids and FAMEs were used as substrates for the enzymatic epoxidation reactions.

**TABLE 1:** Fatty acid (as methyl esters) profile (mg/100 mg oil) of refined rapeseed, sunflower, soybean, and linseed oils, and saturated (SFA), and polyunsaturated (PUFA) totals.

	Vegetable oils			
Fatty acids*	Rapeseed	Sunflower	Soybean	Linseed
C16:0	4.0	7.1	12.7	5.4
C18:0	3.2	4.1	2.1	2.1
C18:1	59.8	31.8	26.6	20.1
C18:2	22.5	56.4	56.3	16.5
C18:3	10.5	0.6	2.2	55.9
*SFA*	7.2	11.2	14.8	7.5
*PUFA*	33.0	57.0	58.5	72.4

*C16:0, palmitic acid; C18:0, stearic acid; C18:1, oleic acid; C18:2, linoleic acid; C18:3, α-linolenic acid.

### UPO Epoxidation of Fatty Acids From Hydrolysis of Different Vegetable Oils

To study the feasibility of enzymatic epoxidation, firstly, hydrolyzates from the four vegetable oils were treated with *Cgl*UPO, *Mro*UPO, and r*Hin*UPO. The conversion degree (which is generally near complete) and the percentages of the different reaction products (Supplementary Figures S3–S5) as well as the epoxidation yields (based on the number of unsaturations of each substrate) were evaluated (Table 2).

**TABLE 2:** Conversion (C, percentage of substrate transformed) of unsaturated fatty acids from oil hydrolyzates (0.1 mM total fatty-acid concentration) by UPOs (100 nM) and relative percentage of products, including monoepoxides, diepoxides, and triepoxides (1E, 2E, and 3E, respectively), and other oxygenated (hydroxy, keto, and carboxy) derivatives (O), and calculated epoxidation yield (EY).

Enzymes	Oils	Products (%)						EY	C
		1E	O-1E	2E	O-2E	3E	O	(%)	(%)
	*Rapeseed*								
*Cgl*UPO	C18:1	90	9	–	–	–	1	99	> 99
	C18:2	13		87				93	> 99
	C18:3	27	–	73	–	–	–	58	> 99
*Mro*UPO	C18:1	12	88^*a*^	–	–	–	–	95	94
	C18:2	–	54	35	11	–	–	72	98
	C18:3	–	36	64	–	–	–	55	> 99
r*Hin*UPO	C18:1	30	69		–	–	1	99	> 99
	C18:2	14	4	80			2	88	99
	C18:3	–	–	> 99	–	–	–	67	> 99
	*Sunflower*								
*Cgl*UPO	C18:1	89	9	–	–	–	2	97	99
	C18:2	16	< 1	83			< 1	91	> 99
	C18:3	–	–	> 99	–	–	–	67	> 99
*Mro*UPO	C18:1	63	32				5	87	92
	C18:2	8	59	22	11	–	–	60	95
	C18:3	–	–	> 99		–	–	67	> 99
r*Hin*UPO	C18:1	17	83	–	–	–	–	97	97
	C18:2	2	5	91			2^*b*^	94	99
	C18:3	–	–	> 99	–	–	–	67	> 99
	*Soybean*								
*Cgl*UPO	C18:1	84	15	–	–	–	1	98	99
	C18:2	9	–	91			< 1	95	99
	C18:3	–	–	> 99	–	–	–	67	> 99
*Mro*UPO	C18:1	46	50	–	–	–	4	90	94
	C18:2	6	57	23	14	–	–	67	98
	C18:3	–	–	> 99	–	–	–	67	> 99
r*Hin*UPO	C18:1	13	87	–	–	–	–	< 95	95
	C18:2	< 1	4	95	–	–	1	95	98
	C18:3	8	–	92	–	–	–	64	> 99
	*Linseed*								
*Cgl*UPO	C18:1	88 (49)	12 (51)	–	–	–	–	> 99 (97)	>99 (97)
	C18:2	8	–	92 (> 99)	–	–	–	96 (66)	> 99 (99)
	C18:3	6	–	94 (91)	–	(9)	–	65 (70)	> 99 (>99)
*Mro*UPO	C18:1	57 (42)	37 (58)^*c*^	–	–	–	6	71 (76)	76 (76)
	C18:2	10 (1)	46 (45)	34 (41)	10 (13)	–	–	70 (75)	97 (98)
	C18:3	8 (< 1)	21 (20)	71 (80)	–	–	–	56 (58)	99 (97)
r*Hin*UPO	C18:1	7	93 (> 99)	–	–	–	–	86 (93)	86 (93)
	C18:2	< 1	3	95 (86)	–	–	2 (14)	92 (85)	95 (99)
	C18:3	< 1	(9)	92 (32)	2 (7)	6 (52)	–	68 (81)	> 99 (>99)

*See chromatographic profiles in Supplementary Figures S6–S9, and chemical structures in Supplementary Figures S3–S5. ^a^Including OH-1E (43%), keto-1E (34%), and COOH-1E (11%). ^b^Including OH (1%) and keto (1%). ^c^Including OH-1E (42%) and keto-1E (16%). Results at 200 nM enzyme in parentheses.*

In the reactions with rapeseed oil hydrolyzate (Supplementary Figure S6), *Cgl*UPO and *Mro*UPO yielded the epoxidized derivatives as main products, including monoepoxide from oleic acid and diepoxides from linoleic and α-linolenic acids. While *Cgl*UPO was remarkably selective toward epoxidation, and yielded the “pure” fatty-acid epoxides, *Mro*UPO formed oxygenated (hydroxy, keto, and carboxy) derivatives of fatty-acid epoxides to a high extent, mainly at the terminal (ω), subterminal (ω-1), and allylic (ω-7) positions of the carbon chain. The higher selectivity of *Cgl*UPO had already been shown with standards of oleic and linoleic acids (Aranda et al., 2018). On the other hand, the unique ability of *Mro*UPO, amongst known UPOs, for oxygenating the terminal position of aliphatic compounds forming carboxylic acids was previously reported (Olmedo et al., 2016). Curiously, the terminal oxygenation of fatty acids was only observed with oleic acid, yielding the dicarboxylic acid epoxide, and not with linoleic and α-linolenic acids. The reaction of r*Hin*UPO with fatty acids is reported here for the first time. Its reactivity toward rapeseed oil hydrolyzate was intermediate between *Cgl*UPO and *Mro*UPO, with the proportion of epoxide and epoxidized derivatives from oleic acid being similar to those found with *Mro*UPO. However, an epoxidation pattern closer to *Cgl*UPO was observed with linoleic and α-linolenic acids except for the diastereoselectivity of linoleic acid diepoxides where the *anti* isomer was predominantly formed, as in the *Mro*UPO reaction. Moreover, predominant hydroxylation of oleic acid in ω-6 (instead of allylic or subterminal positions) was observed. The high selectivity of the above UPOs epoxidizing oleic acid (up to 100%) differs from that of P450 (BM3) where hydroxylation (> 97%) predominates over epoxidation (< 3%), with a substrate conversion around 80%.

In reactions with sunflower and soybean oil hydrolyzates (Table 2 and Supplementary Figures S7, S8), both with a predominance of linoleic acid, *Cgl*UPO, and *Mro*UPO yielded monoepoxides of oleic and linoleic acids, and diepoxides of linoleic and α-linolenic acids. Similar to the reactions with rapeseed oil hydrolyzate, *Cgl*UPO was more selective toward “pure” epoxidation than *Mro*UPO, and formed mainly the oleic acid epoxide and the linoleic acid diepoxides (with the *syn* diastereoisomer predominating). *Mro*UPO, in addition to pure epoxides, formed their further oxygenated derivatives, mainly at the allylic (ω-7) position. Respecting stereoselectivity of linoleic acid diepoxides, the *anti*-isomer was preferably formed by *Mro*UPO. The r*Hin*UPO reaction pattern followed that of rapeseed oil, since oleic acid was almost fully epoxidized, and high amounts of hydroxy epoxides (especially at ω-6) were observed. Likewise, diepoxides from linoleic and α-linolenic acids predominated over monoepoxides. Moreover, with both *Mro*UPO and r*Hin*UPO, the *anti*-isomer of linoleic acid diepoxides was preferably formed.

**FIGURE 1:**
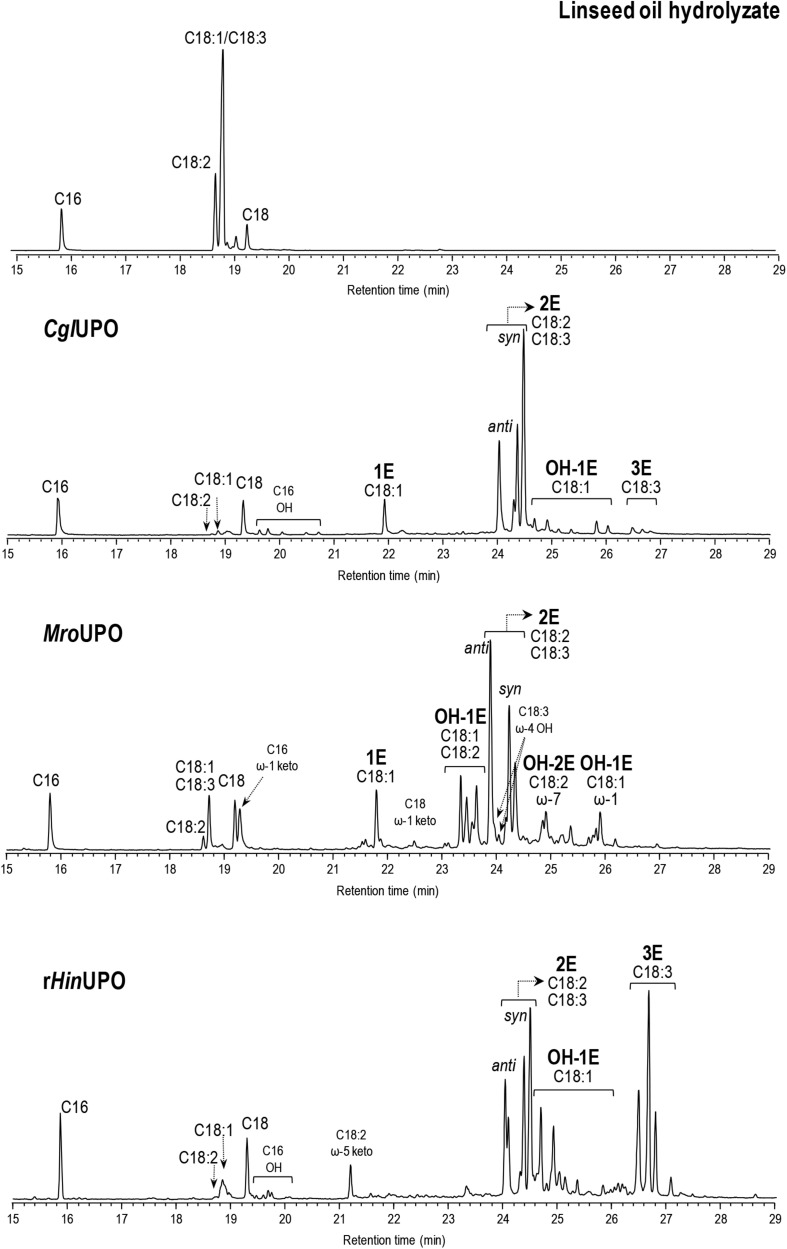
GC-MS analysis of linseed oil hydrolyzate (0.1 mM total fatty-acid concentration) reactions with *Cgl*UPO, *Mro*UPO, and r*Hin*UPO (200 nM), showing monoepoxides, diepoxides (including *syn* and *anti* isomers from linoleic acid), and triepoxides (1E, 2E, and 3E, respectively), and other oxygenated (hydroxy, OH, and keto) derivatives. Control chromatogram is also shown (in the top). The oleic and α-linolenic peaks partially overlap under the optimized conditions for analysis of their oxygenated products, and quantitation was done using specific ions.

The reactions of linseed oil hydrolyzate are particularly interesting due to the high α-linolenic acid content (56%) in this oil, and the subsequent possibility of obtaining triepoxides, which are promising building blocks and cross-linking agents difficult to be synthesized (Çelik et al., 2005; Sauveplane et al., 2009). The three enzymes revealed an oxygenation pattern (Table 2 and Supplementary Figure S9) similar to that obtained with sunflower and soybean oils, including monoepoxides (preferably from oleic acid) and diepoxides (from linoleic and α-linolenic acids). Additionally, hydroxylated derivatives at ω-7 and ω-6 (by *Mro*UPO and r*Hin*UPO, respectively), of epoxidized oleic acid, and hydroxylated derivatives at ω-7 of mono- and di-epoxidized linoleic acid at (by *Mro*UPO) were formed. Interestingly, some triepoxides (6% of total products) were produced by r*Hin*UPO under the reaction conditions used (including 100 nM enzyme). Moreover, increasing the r*Hin*UPO loading (to 200 nM) allowed to increase the triepoxide yield up to 52% (Figure 1 and Table 2), an interesting result for the industrial use of these biocatalysts that is further explored in the upscaling section included below. The formation of fatty-acid triepoxides by UPOs is reported here for the first time.

In summary, although the three UPOs showed similar epoxidation yields toward oleic acid, *Cgl*UPO yielded more epoxides from linoleic acid, and r*Hin*UPO from α-linolenic acid (Table 2). Concerning saturated fatty acids, which represent a minor fraction of compounds in vegetable oils (7–15% in Table 1), they were poorly transformed by these UPOs (only up to 56%) (Supplementary Figures S6–S9). Focusing on products, partially regioselective oxygenation (at ω-1) was only observed with *Mro*UPO, especially with palmitic acid, while unspecific hydroxylation occurred with the other two UPOs.

### UPO Epoxidation of FAMEs From Transesterification of Different Vegetable Oils

In addition to the hydrolyzates, the transesterified oils were also tested as substrates of the three UPOs to evaluate their epoxidation feasibility. The conversion degrees of the different FAMEs and the different reaction products (Supplementary Figures S3–S5), as well as the epoxidation yields were evaluated (Table 3) revealing first that higher enzyme doses (of all UPOs) were needed to achieve similar conversion degrees to those obtained with the oil hydrolyzates.

**TABLE 3:** Conversion (C, percentage of substrate transformed) of unsaturated FAME (0.1 mM total concentration) from transesterified oils by UPO (0.5 μM) and relative percentage of products (methyl esters), including mono-, di-, and tri-epoxides (1E, 2E, and 3E, respectively), and other oxygenated (hydroxy and keto) derivatives (O), and calculated epoxidation yield (EY).

Enzyme	Oils	Products (%)						EY	C
		1E	O-1E	2E	O-2E	3E	O	(%)	(%)
	*Rapeseed*								
*Cgl*UPO	C18:1	91	9	–	–	–	< 1	92	92
	C18:2	–	7	93	–	–	–	95	98
	C18:3	–	–	> 99	–	–	–	67	> 99
*Mro*UPO	C18:1	61	38	–	–	–	< 1	91	91
	C18:2	12	33	32	23	–	–	77	99
	C18:3	–	–	> 99	–	–	–	67	> 99
r*Hin*UPO	C18:1	54	46	–	–	–	–	91	91
	C18:2	–	10	90	–	–	–	92	97
	C18:3	–	–	66	10	24	–	71	95
	*Sunflower*								
*Cgl*UPO	C18:1	97	–	–	–	–	3	95	99
	C18:2	40	2	58	–	–	–	79	> 99
	C18:3	–	–	> 99	–	–	–	67	> 99
*Mro*UPO	C18:1	79	21	–	–	–	–	82	82
	C18:2	15	21	50	14	–	–	70	85
	C18:3	–	–	> 99	–	–	–	67	> 99
r*Hin*UPO	C18:1	54	46^*a*^	–	–	–	–	> 99	> 99
	C18:2	–	14	86	–	–	–	93	> 99
	C18:3	–	–	> 99	–	–	–	67	> 99
	*Soybean*								
*Cgl*UPO	C18:1	94	3	–	–	–	3	94	97
	C18:2	22	3	75	–	–	–	87	99
	C18:3	–	–	> 99	–	–	–	67	> 99
*Mro*UPO	C18:1	91	7	–	–	–	2	78	80
	C18:2	8	24	62	6	–	–	77	89
	C18:3	–	–	> 99	–	–	–	67	> 99
r*Hin*UPO	C18:1	64	36^*b*^	–	–	–	–	> 99	> 99
	C18:2	–	8	89	3	–	–	96	> 99
	C18:3	–	–	73	–	28	–	76	> 99
	*Linseed*								
*Cgl*UPO	C18:1	94 (68)	5 (32)	–	–	–	1 (< 1)	94 (91)	95 (91)
	C18:2	–	6 (4)	91 (96)	–	–	3	90 (95)	96 (97)
	C18:3	3 (< 1)	6 (6)	85 (64)	–	6 (30)	< 1	65 (73)	98 (98)
*Mro*UPO	C18:1	87 (80)	13 (20)	–	–	–		95 (91)	95 (91)
	C18:2	19	42 (31)	26 (24)	13 (45)	–	–	67 (82)	98 (97)
	C18:3		18 (6)	78 (83)	1 (2)	3 (9)		61 (66)	98 (97)
r*Hin*UPO	C18:1	46 (44)	54^*c*^ (56)^*d*^	–	–	–	–	> 99 (> 99)	> 99 (> 99)
	C18:2	–	6 (7)	94 (93)	–	–	–	< 97 (97)	> 99 (> 99)
	C18:3	–	10 (10)	52 (51)	13 (11)	26 (28)	–	72 (73)	> 99 (> 99)

*See chromatographic profiles in Supplementary Figures S10–S13, and chemical structures in Supplementary Figures S3–S5.^a^Including OH-1E (38%) and keto-1E (8%). ^b^Including OH-1E (35%) and keto-1E (1%). ^c^Including OH-1E (41%) and keto-1E (13%). ^d^Including OH-1E (46%) and keto-1E (10%). Results at 1 μM enzyme in parentheses.*

The *Cgl*UPO behavior was similar to that observed with the oil hydrolyzates, that is, a remarkable selectivity toward “pure” epoxidation, producing the monoepoxidation of oleic acid and the diepoxidation of linoleic and α-linolenic methyl esters (Supplementary Figures S10–S13). Moreover, *Mro*UPO showed improved selectivity toward pure epoxidation of methyl oleate and linoleate (particularly in diepoxides) compared with their saponified counterparts. This led to lower amounts of hydroxylated derivatives of mono- and diepoxides, although a new hydroxylated epoxide from methyl oleate (at ω-10) was formed by *Mro*UPO. Furthermore, unlike in hydrolyzate reactions, terminal hydroxylation was not observed with FAMEs. Likewise, the improved pure epoxidation of methyl oleate (compared with oleic acid) was also observed in the r*Hin*UPO reactions.

**FIGURE 2:**
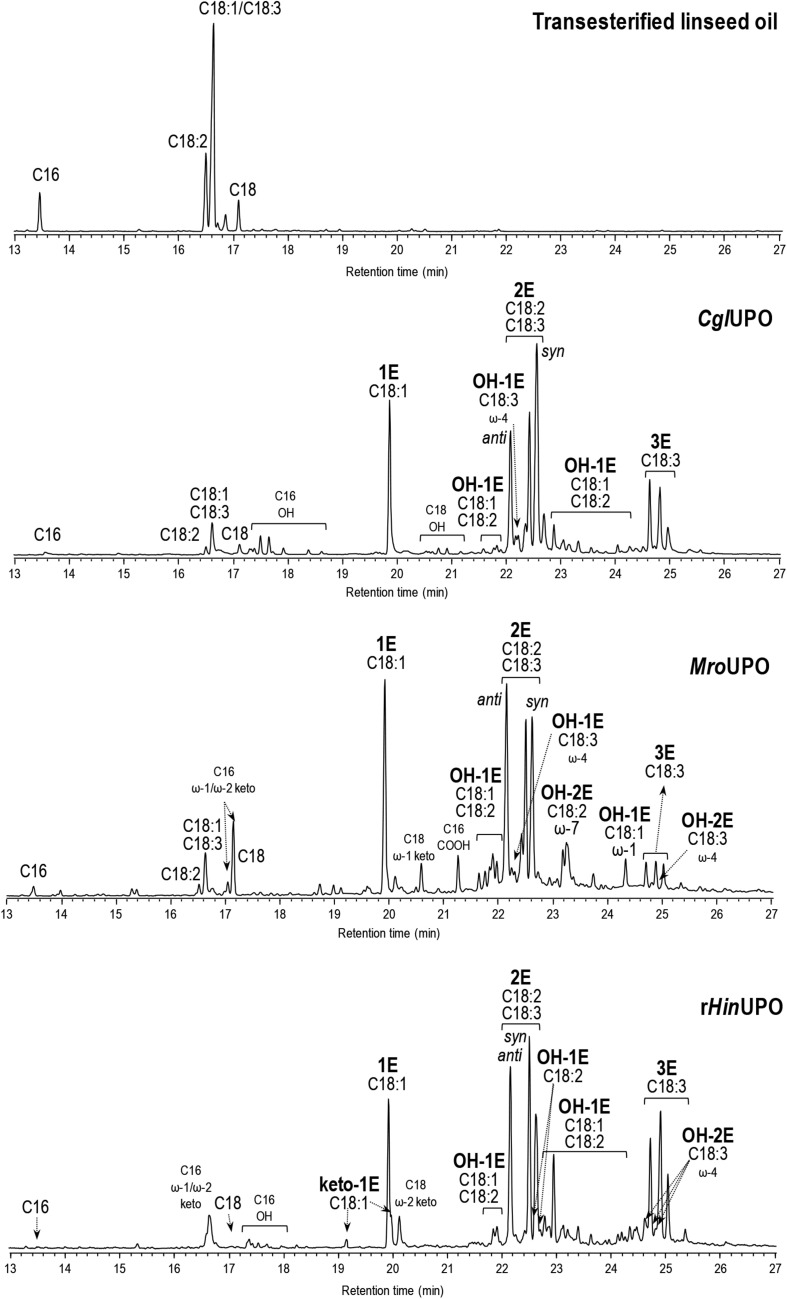
GC-MS analysis of transesterified linseed oil (0.1 mM total fatty-acid concentration) reactions with *Cgl*UPO, *Mro*UPO, and r*Hin*UPO (1 μM), showing monoepoxides, diepoxides (including *syn* and *anti* isomers from linoleic acid) and triepoxides (1E, 2E, and 3E, respectively), and other oxygenated (hydroxy, OH, and keto) derivatives. Control chromatogram is also shown (in the top).

Triepoxides were formed in the r*Hin*UPO reactions with linseed oil FAME in higher amount (up to 26%) than with the linseed oil hydrolyzate. Interestingly, triepoxides were also observed in the *Cgl*UPO (6%) and *Mro*UPO (3%) reactions with transesterified linseed oil, and in the r*Hin*UPO reactions with transesterified rapeseed and soybean oils up to 76% epoxidation yield (Supplementary Figure S13). With the aim of increasing the production of FAME triepoxides, reactions with twofold enzyme dose (1 μM) were conducted with the three enzymes (Figure 2) and higher amounts of triepoxides were obtained with *Cgl*UPO (up to 30%) and *Mro*UPO (up to 9%) improving their epoxidation yields (from 65 to 73%, and from 61 to 66%, respectively) (Table 3).

Finally, the enzyme behavior with the saturated FAMEs was dissimilar (Figure 2 and Supplementary Figures S10–S13). *Cgl*UPO and *Mro*UPO reached moderate to good conversions, while r*Hin*UPO achieved quantitative conversions. Regarding the reaction products, *Cgl*UPO gave a series of hydroxylated compounds (from ω-8 to ω-3 positions) while terminal and/or subterminal oxygenation was observed with r*Hin*UPO and *Mro*UPO. In the latter case, the carboxylic acid and the (ω-1) ketone predominated. With r*Hin*UPO, the (ω-2/ω-1) ketones were obtained with very high regioselectivity.

### Upscaling Epoxidation of Oil Fatty Acids by UPO

Aimed to scaling-up the production of epoxidized fatty acids for industrial applicability, an attempt of increasing the substrate loading of the enzymatic reactions was performed. With this purpose, sunflower oil was selected considering economical, technical suitability, and sustainability aspects. For comparative purposes, the substrate/enzyme ratio of the previous reactions (S/E 1000) was fixed, remaining as reaction variables the substrate loading, the reaction time and the H_2_O_2_ dose. Besides, taking into account the pH effect on fatty-acid solubility and enzyme activity, the reactions were performed at pH 7.0, which enabled to increase the substrate concentration up to 300-fold (30 mM substrate) using 40% (v/v) of acetone. Moreover, different reaction times (1–20 h) and H_2_O_2_ doses (1–8 equiv per unsaturation) were assayed to attain the best conversion and epoxidation degrees.

With *Mro*UPO, conversions over 98% of linoleic and α-linolenic acids, and 77% of oleic acid were reached (Table 4 and Supplementary Figure S14) within 2.5 h, using of 93.5 mM H_2_O_2_ (i.e., 2.1 equiv per unsaturation). Under these conditions, a decrease in the epoxidation yield was observed compared with the initial reactions, together with higher amounts of hydroxylated derivatives (23 and 6% from oleic and linoleic acids, respectively), and a reduction of diepoxides. These differences are explained by the lower activity of *Mro*UPO at pH 7.0 (compared with optimal pH 5.5). To check the effect of pH, we also performed the *Mro*UPO reaction at pH 5.5, maintaining the other conditions. At this pH (and 4 mM substrate loading) the conversion rates were higher and close to quantitative (Table 4). In particular, a general increment in the epoxidation was observed for all compounds, with higher amounts of diepoxides from linoleic and α-linolenic acids.

**TABLE 4:** Conversion (C, percentage of substrate transformed) of unsaturated fatty acids from upscaled treatment of sunflower oil hydrolyzate (30 mM total fatty-acid concentration, and pH 7 unless otherwise stated by several UPO (30 μM), at different reaction times 1 h for *Cgl*UPO and r*Hin*UPO and 2.5 h for *Mro*UPO) and relative percentage of reaction products, including mono-, di-, and tri-epoxides (1E, 2E, and 3E, respectively), and other oxygenated (hydroxyl and keto) derivatives (O), and calculated epoxidation yield (EY).

Enzymes	Fatty acids	Products (%)					EY	C
		1E	O-1E	2E	O-2E	O	(%)	(%)
*Cgl*UPO	C18:1	77	22	–	–	1	99	> 99
	C18:2	–	17^*a*^	84	–	–	93	> 99
	C18:3	–	–	> 99	–	–	67	> 99
*Mro*UPO	C18:1	72 (71)	5 (16)	–	–	23 (13)	59 (87)	77 (> 99)
	C18:2	69 (35)	21 (33)	4 (22)	(3)	6 (8)	48 (59)	98 (> 99)
	C18:3	> 99		(> 99)	–	–	33 (67)	> 99 (> 99)
r*Hin*UPO	C18:1	68	32	–	–	–	99	99
	C18:2	–	6^*b*^	94	–	–	97	> 99
	C18:3	–	–	> 99	–	–	67	> 99

*See chromatographic profiles in Supplementary Figure S14, and chemical structures in Supplementary Figures S3–S5.^a^Including OH-1E (4%) and keto-1E (13%). ^b^Including OH-1E (3%) and keto-1E (3%). Results with 4 mM substrate and pH 5.5, are shown in parentheses.*

Interestingly, in the *Cgl*UPO and r*Hin*UPO reactions with 30 mM substrate loading (Table 4 and Supplementary Figure S14), conversions over 99% of all unsaturated fatty acids were produced after 1 h with 240.0 mM (5.5 equiv) and 93.5 mM (2.1 equiv) H_2_O_2_ concentration, respectively (while longer reaction times were required with *Mro*UPO). The H_2_O_2_ concentration in these reactions was over-stoichiometric (2.1–5.5 equiv) to overcome the “catalase-like” activity produced by the reaction of peroxide-activated UPO with H_2_O_2_ (Karich et al., 2016). Although more hydroxy/keto epoxides were found with *Cgl*UPO, compared with initial conditions, the opposite happened in the r*Hin*UPO reactions, in which a strong increase of the desired pure epoxide of oleic acid (from 17 to 68%) was produced.

## Conclusion

A series of oil-producing plants of world-wide significance are available for the production of renewable lipid epoxides and other oxygenated derivatives. Commercially exploited oil seeds, such as rapeseed, soybean, sunflower, or linseed, exhibit a considerable variation in their fatty acid profiles, which makes them interesting raw materials for production of different lipid compounds. The hydrolyzated and transesterified products of the above vegetable oils were treated with three fungal UPOs to obtain epoxides. The three enzymes were capable of transforming the fatty acids and FAMEs from the oils into the corresponding epoxide derivatives, although some significant differences in selectivity toward epoxidation were observed, with *Cgl*UPO being generally more selective. Noteworthy is the ability of these UPOs, particularly r*Hin*UPO, to produce triepoxides from these samples. Therefore, UPOs appear as promising biocatalysts for the environmentally friendly production of reactive fatty-acid epoxides given their self-sufficient monooxygenase activity with high epoxidation selectivity, including recently reported enantioselectivity (in addition to strict regioselectivity) of some of their reactions (Municoy et al., 2020). However, in spite of all recent progresses in our understanding of UPO catalysis and application (Wang et al., 2017; Hofrichter et al., 2020), some difficulties are still to be solved, such as the inactivation by H_2_O_2_ that affects enzyme reuse. The latter could be overcome by continuous feeding low H_2_O_2_ concentration, or its *in situ* generation by enzymatic or chemical systems, enabling to further increase the concentration of FA substrates and final epoxide products.

## Data Availability Statement

The original contributions presented in the study are included in the article/Supplementary Material, further inquiries can be directed to the corresponding author/s.

## Author Contributions

AG-B: methodology development, oil saponification, transesterification, enzymatic treatments, product analysis, and first draft writing. GM: contribution to chemical characterization of the oils. OH-M: production of r*Hin*UPO. JK and KS: production of *Mro*UPO and *Cgl*UPO. JdR: contribution to mass-spectrometry analyses. AM: advise in enzymatic treatment and contribution to final draft writing. AG: work design and supervision, funding acquisition, and contribution to first and final draft writing. All authors contributed to the article and approved the version submitted.

## Conflict of Interest

JK and KS were employed by the company JenaBios GmbH, and OH-M by Novozymes A/S. The remaining authors declare that the research was conducted in the absence of any commercial or financial relationships that could be construed as a potential conflict of interest.

## Supplementary Material

Supplementary figures show: (i–ii) GC-MS analyses of intact oils and different linseed-oil samples; (iii–v) chemical structures of oleic, linoleic, and α-linolenic acids, and their oxygenated derivatives; (vi–ix) GC-MS analyses of oil-hydrolyzate reactions; (x–xiii) GC-MS analysis of transesterified-oil reactions; and (xiv) GC-MS analysis of upscaled reactions of sunflower-oil hydrolyzate.
