# Supplementary material for: High Epoxidation Yields of Vegetable Oil Hydrolyzates and Methyl Esters by Selected Fungal Peroxygenases
Source: Front Bioeng Biotechnol. 2021 Jan 5;8:605854. doi: 10.3389/fbioe.2020.605854 (PMC7813931; doi:10.3389/fbioe.2020.605854)
Supplement: Supplementary file 1 [file Data_Sheet_1.pdf]

Supplementary Material (FILE S1)

High Epoxidation Yields of Vegetable Oil Hydrolyzates and Methyl Esters by Selected Fungal Peroxygenases

Alejandro González-Benjumea[1], Gisela Marques[1], Owik M. Herold-Majumdar[2], Jan Kiebist[3], Katrin Scheibner[3], José C. del Río[1], Angel T. Martínez[4] and Ana Gutiérrez[1]*

[1] Instituto de Recursos Naturales y Agrobiología de Sevilla (IRNAS), CSIC, Av. Reina Mercedes 10, E-41012 Seville, Spain, [2] Novozymes A/S, Krogshoejvej 36, 2880 Bagsvaerd, Denmark ,[3] JenaBios GmbH, Löbstedter Str. 80, 07749 Jena, Germany,[4] Centro de Investigaciones Biológicas Margarita Salas (CIB), CSIC, Ramiro de Maeztu 9, E-28040 Madrid, Spain

This Supplementary **File S1** includes GC-MS analysis of intact oils **(Figure S1**), GC-MS analysis of different linseed-oil samples (**Figure S2**), chemical structures of oleic acid and its oxygenated derivatives (**Figure S3**), chemical structures of linoleic acid and its oxygenated derivatives (**Figure S4**), chemical structures of α-linolenic acid and its oxygenated derivatives (**Figure S5**), GC-MS analysis of reactions of rapeseed-oil hydrolyzate (**Figure S6**), GC-MS analysis of reactions of sunflower-oil hydrolyzate (**Figure S7**), GC-MS analysis of reactions of soybean-oil hydrolyzate (**Figure S8**), GC-MS analysis of reactions of linseed-oil hydrolyzate (**Figure S9**), GC-MS analysis of reactions of transesterified rapeseed oil (**Figure S10**), GC-MS analysis of reactions of transesterified sunflower oil (**Figure S11**), GC-MS analysis of reactions of transesterified soybean oil (**Figure S12**), GC-MS analysis of reactions of transesterified linseed oil (**Figure S13**), and GC-MS analysis of upscaled reactions of sunflower-oil hydrolyzate (**Figure S14**).

* **Correspondence**: Ana Gutiérrez (anagu@irnase.csic.es)

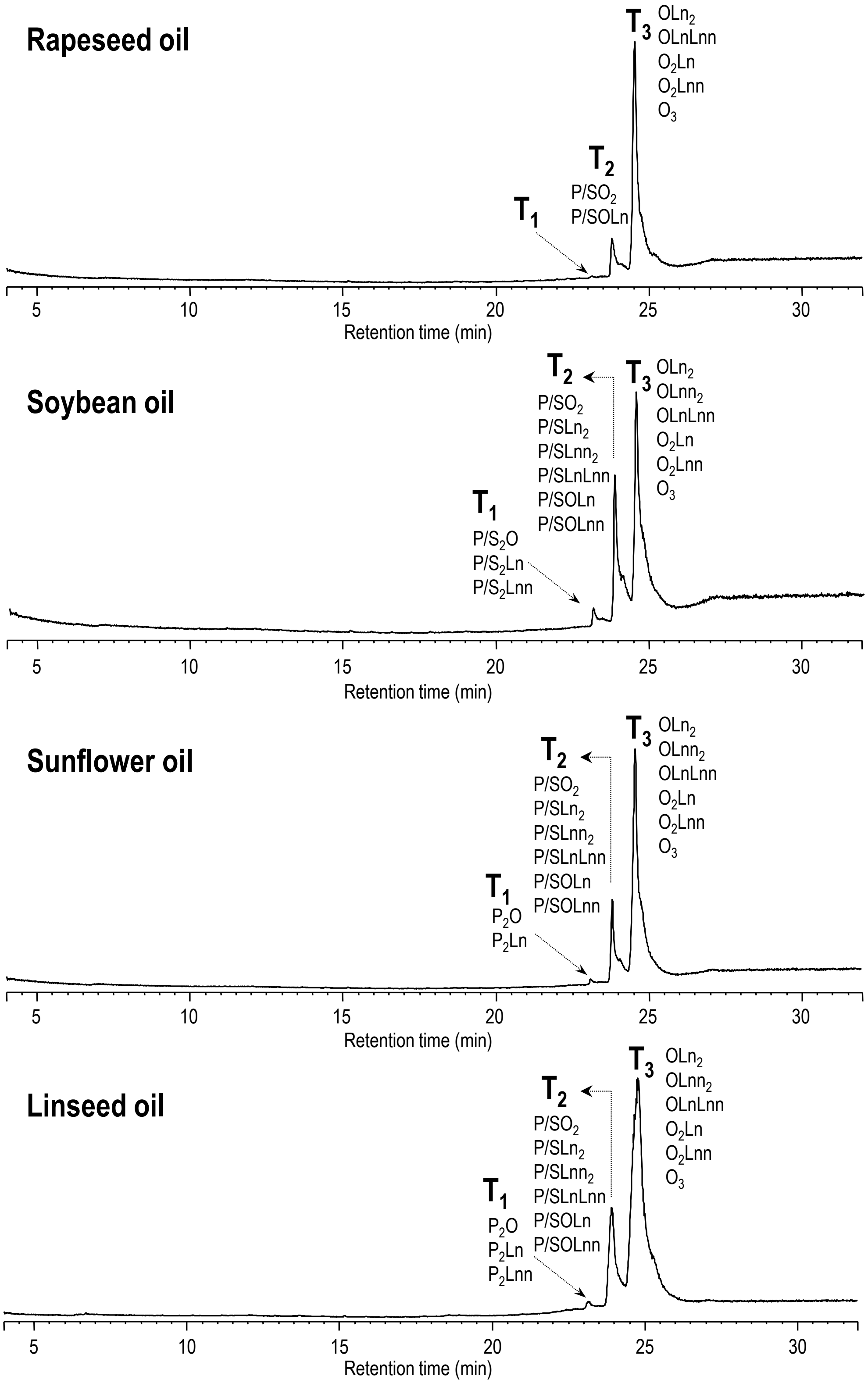

Supplementary Figure S1. GC-MS analysis of “intact” oils showing the fatty acid composition of triglycerides containing one (**T1**), two (**T2**) and three (**T3**) unsaturated fatty acids, in rapeseed, soybean, sunflower and linseed oils. Fatty acid abbreviations: P, palmitic acid; S, stearic acid; O, oleic acid; Ln, linoleic acid; and Lnn, α-linolenic acid.

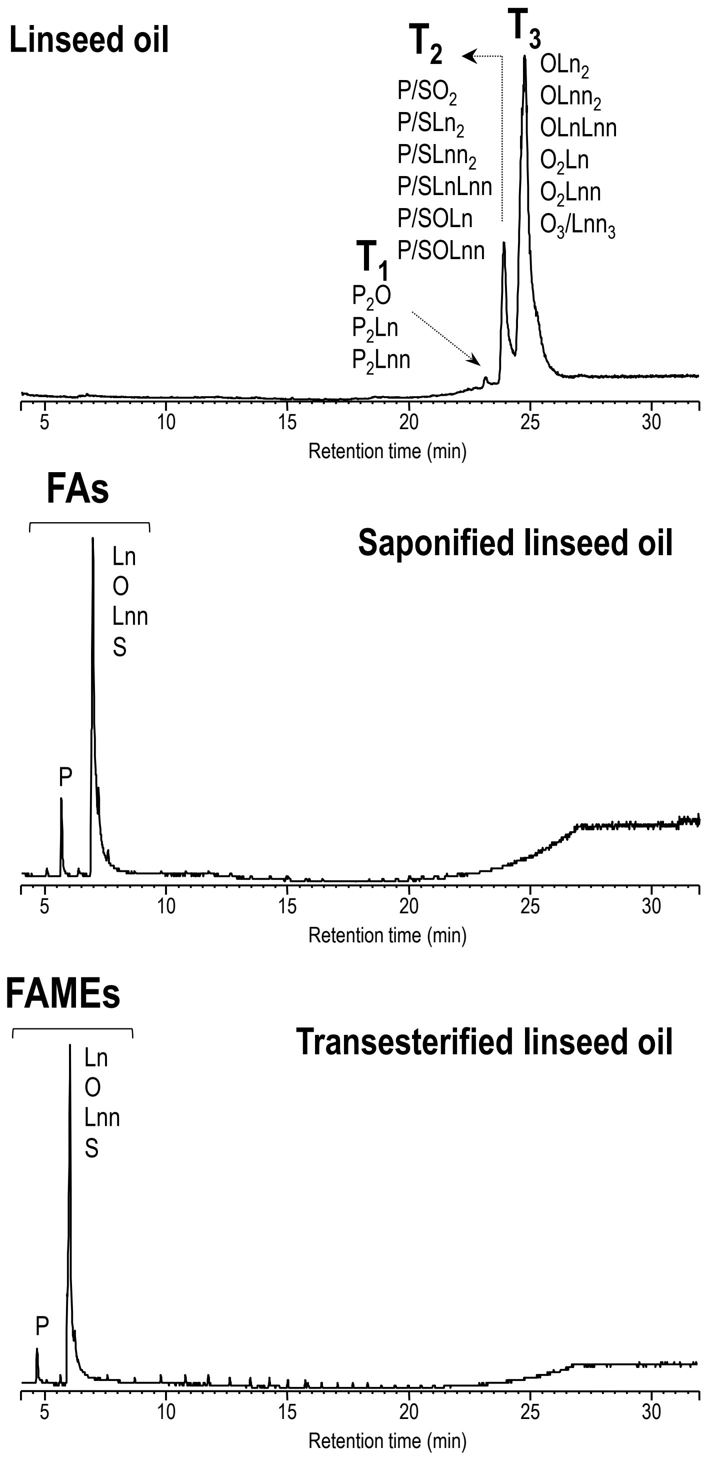

Supplementary Figure S2. GC-MS analysis of different linseed-oil samples including: **i)** "intact" oil with its triglyceride composition (as in **Figure S1**); **ii)** saponifed oil; and **iii)** transesterified oil; n denotes the number of unsaturated fatty acids in triglyceride; P: palmitic acid, S: stearic acid, O: oleic acid, Ln: linoleic acid, Lnn: α-linolenic acid.

OLEIC ACID

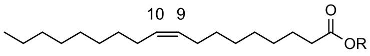

PRODUCTS

Epoxides

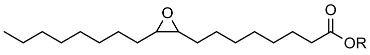

9-Epoxide

Hydroxylated derivatives of 9-Epoxide

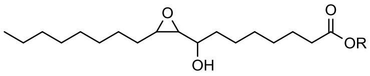

(ω-10)-OH

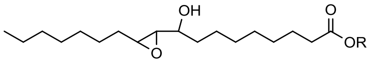

(ω-9)-OH

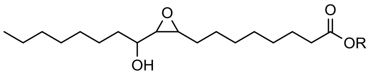

(ω-7)-OH

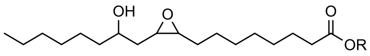

(ω-6)-OH

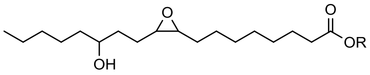

(ω-5)-OH

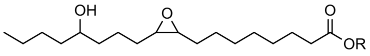

(ω-4)-OH

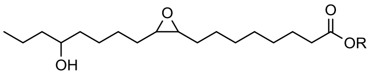

(ω-3)-OH

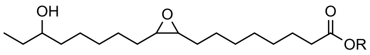

(ω-2)-OH

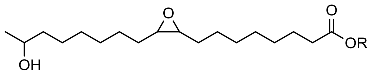

(ω-1)-OH

Carbonyl derivatives of 9-Epoxide

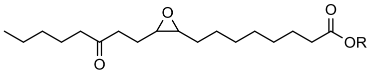

(ω-5)-keto

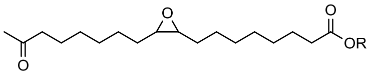

(ω-1)-keto

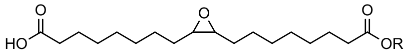

COOH

Hydroxylated derivatives of oleic acid

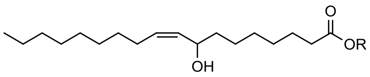

(ω-10)-OH

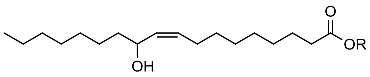

(ω-7)-OH

Supplementary Figure S3. Chemical structures of oleic acid and its oxygenated derivatives from UPO reactions with saponified (chromatograms in **Figures 1**, **S6-S9** and **S14**) and transesterified (chromatograms in **Figures 3** and **S10-S13**) vegetable oils (the latter as methyl esters).

LINOLEIC ACID

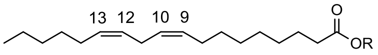

PRODUCTS

mono-Epoxides

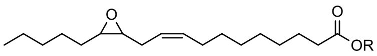

12,13-Epoxide

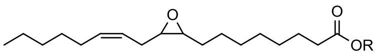

9,10-Epoxide

Hydroxylated derivatives of mono-Epoxides

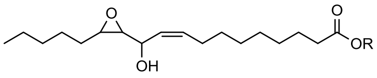

(ω-7)-OH-12E

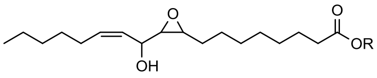

(ω-7)-OH-9E

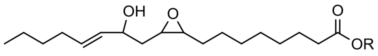

(ω-6)-OH-9E

di-Epoxides

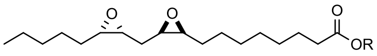

anti

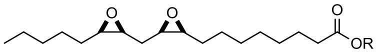

syn

Hydroxylated derivatives of di-Epoxides

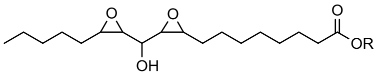

(ω-7)-OH

Hydroxylated derivatives of linoleic acid

(ω-9)-OH

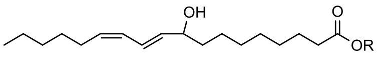

(ω-4)-OH

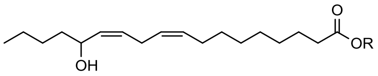

Supplementary Figure S4. Chemical structures of linoleic acid and its oxygenated derivatives from UPO reactions with saponified (chromatograms in **Figures 1**, **S6-S9** and **S14**) and transesterified (chromatograms in **Figures 3** and **S10-S13**) vegetable oils (the latter as methyl esters).

α-LINOLENIC ACID

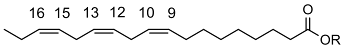

PRODUCTS

mono-Epoxides

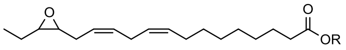

15,16-Epoxide

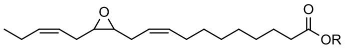

12,13-Epoxide

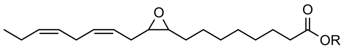

9,10-Epoxide

Hydroxylated derivatives of mono-Epoxides

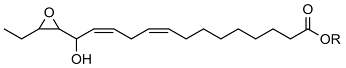

(ω-4)-OH-15E

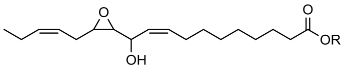

(ω-7)-OH-12E

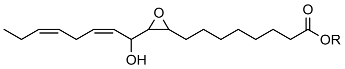

(ω-7)-OH-9E

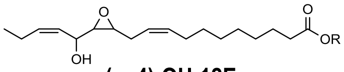

(ω-4)-OH-12E

di-Epoxides

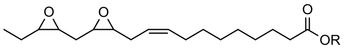

12,13-15,16-di-Epoxide

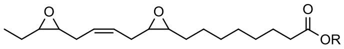

9,10-15,16-di-Epoxide

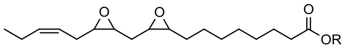

9,10-12,13-di-Epoxide

Hydroxylated derivatives of di-Epoxides

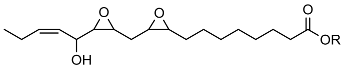

(ω-4)-OH-di-Epoxide

tri-Epoxides

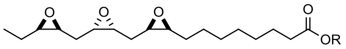

anti

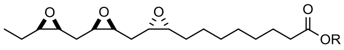

anti/*syn*

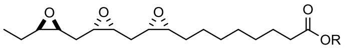

syn/*anti*

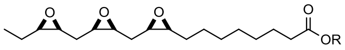

syn

Hydroxylated derivatives of linolenic acid

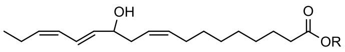

(ω-6)-OH

Supplementary Figure S5. Chemical structures of α-linolenic acid and its oxygenated derivatives from UPO reactions with saponified (chromatograms in **Figures 1**, **S6-S9** and **S14**) and transesterified (chromatograms in **Figures 3** and **S10-S13**) vegetable oils (the latter as methyl esters).

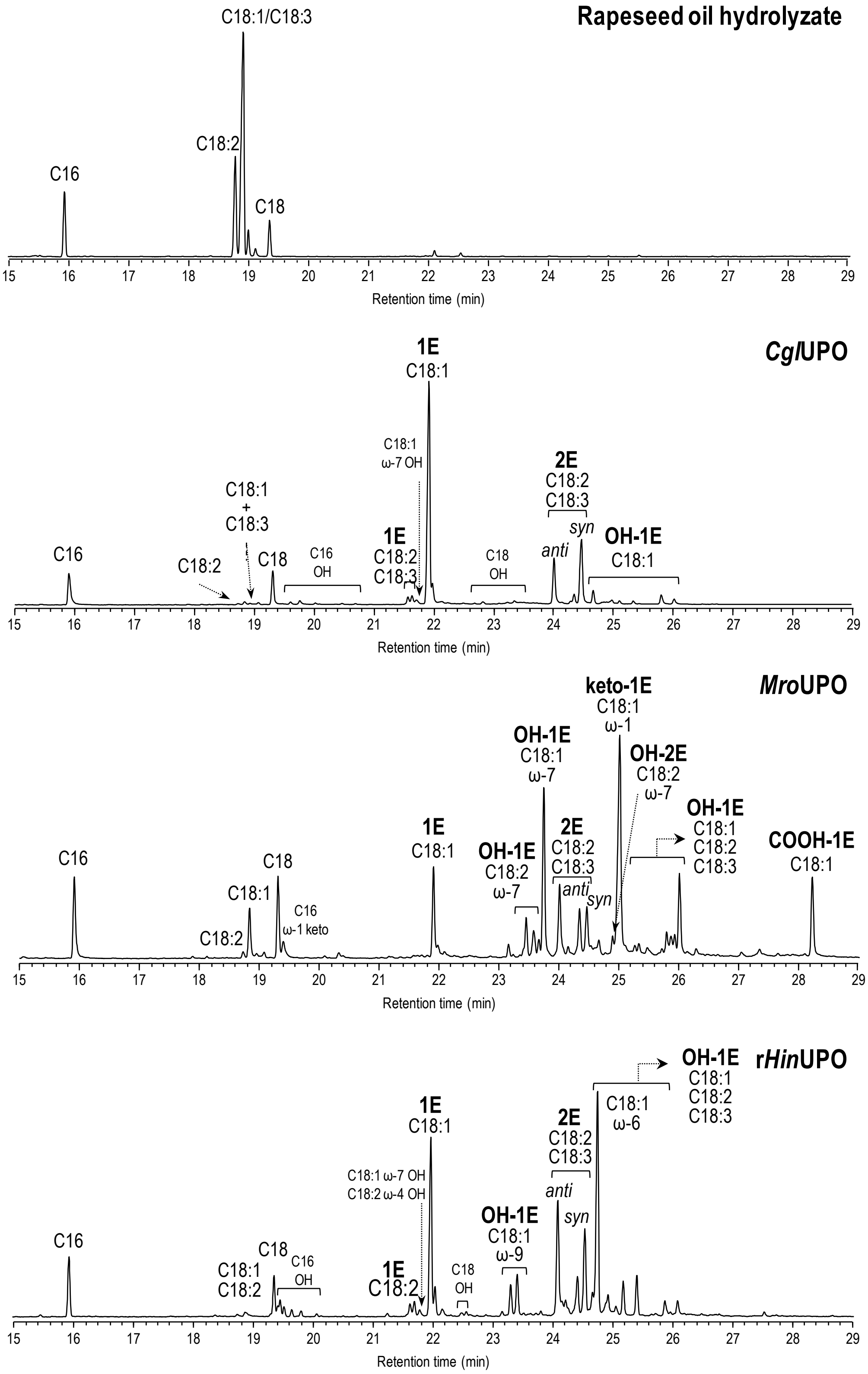

Supplementary Figure S6. GC-MS analysis of rapeseed oil hydrolyzate (0.1 mM total fatty-acid concentration) reactions with *Cgl*UPO, *Mro*UPO and r*Hin*UPO (100 nM), showing monoepoxides and diepoxides (including *syn* and *anti* isomers from linoleic acid) (1E and 2E, respectively) and other oxygenated (hydroxy, OH; keto; and carboxy, COOH) derivatives (often combined in the same compound). Control chromatogram is also shown in the top.

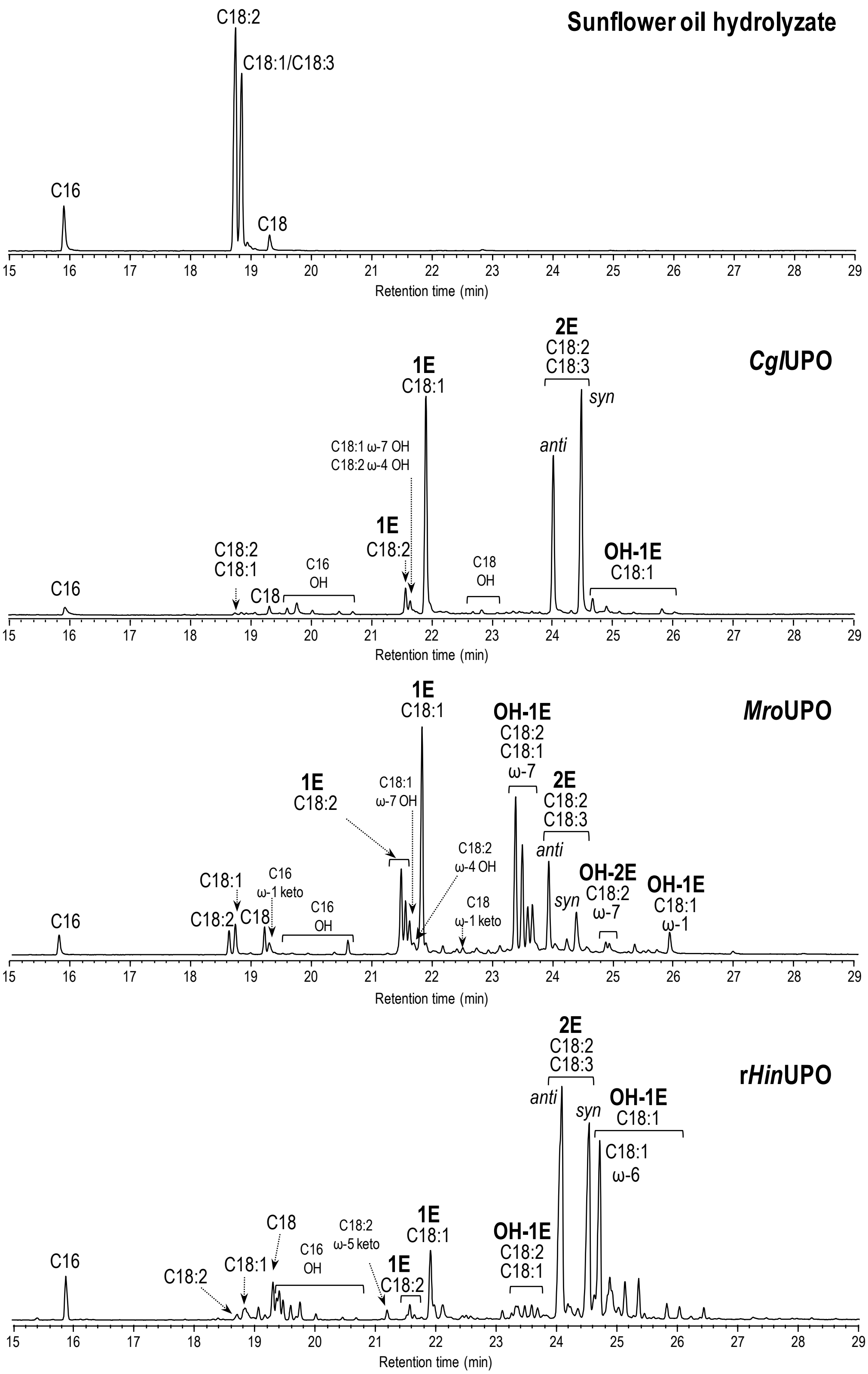

Supplementary Figure S7. GC-MS analysis of reactions of sunflower oil hydrolyzate with *Cgl*UPO, *Mro*UPO and r*Hin*UPO (100 nM), showing monoepoxides and diepoxides (including *syn* and *anti* isomers from linoleic acid; 1E and 2E, respectively) and other oxygenated (hydroxy, OH; and keto) derivatives. Control chromatogram is also shown in the top.

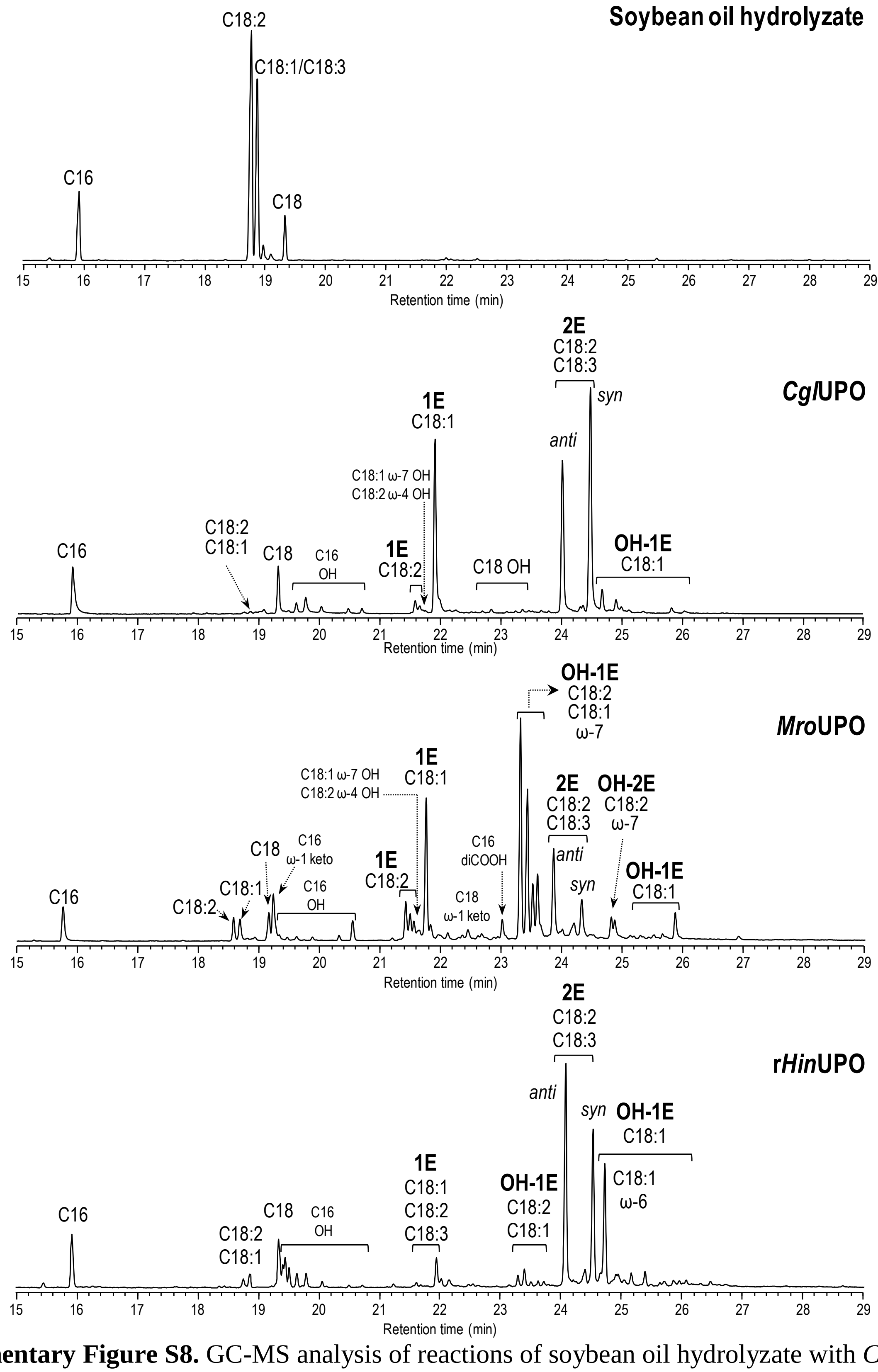

Supplementary Figure S8. GC-MS analysis of reactions of soybean oil hydrolyzate with *Cgl*UPO, *Mro*UPO and r*Hin*UPO (100 nM), showing monoepoxides and diepoxides (including *syn* and *anti* isomers from linoleic acid; 1E and 2E, respectively) and other oxygenated (hydroxy, OH; keto; and carboxy, COOH) derivatives. Control chromatogram is also shown in the top.

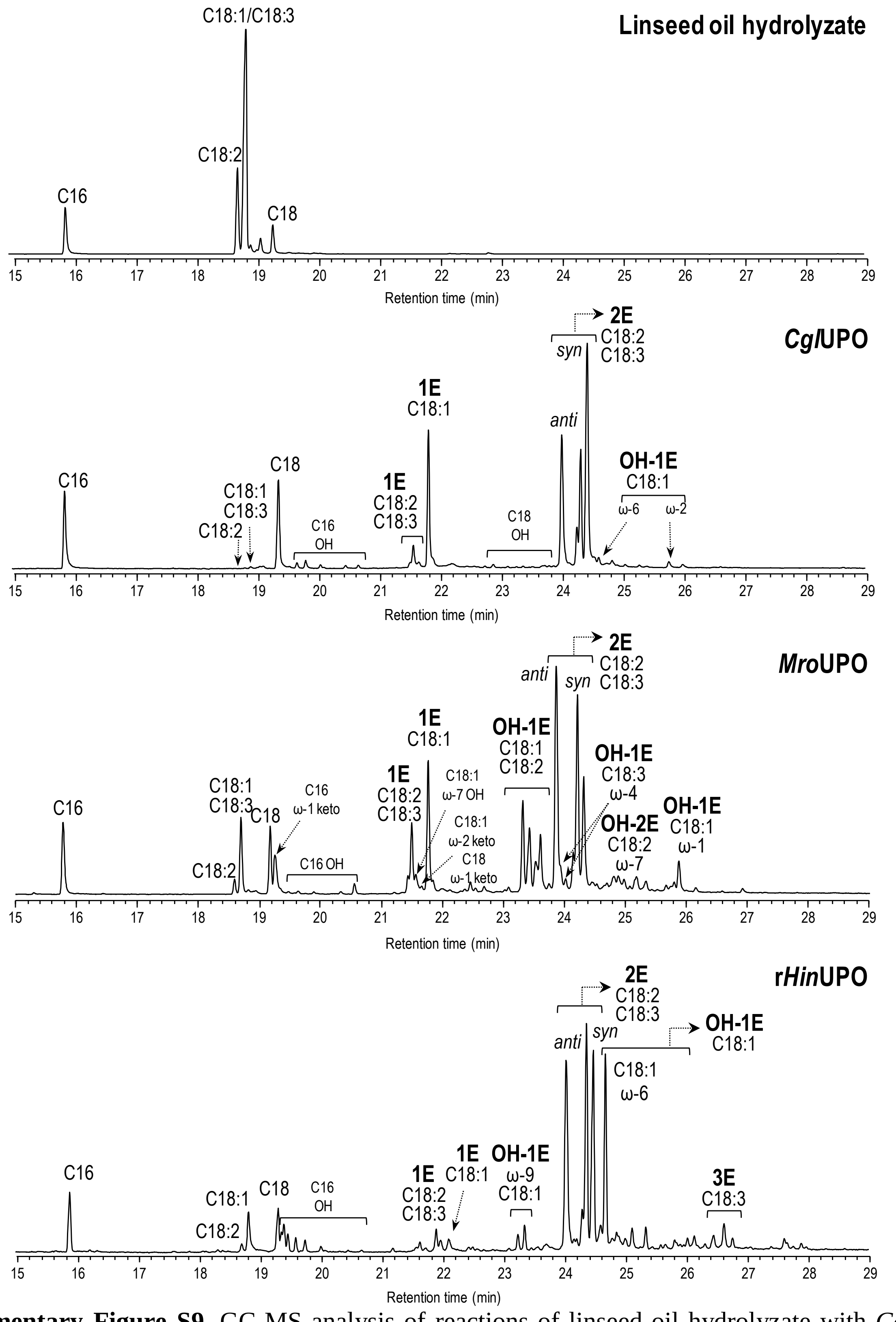

Supplementary Figure S9. GC-MS analysis of reactions of linseed oil hydrolyzate with *Cgl*UPO, *Mro*UPO and r*Hin*UPO (100 nM), showing monoepoxides, diepoxides (including *syn* and *anti* isomers from linoleic acid) and triepoxides (1E, 2E and 3E, respectively) and other oxygenated (hydroxy, OH; and keto) derivatives. Control chromatogram is also shown in the top.

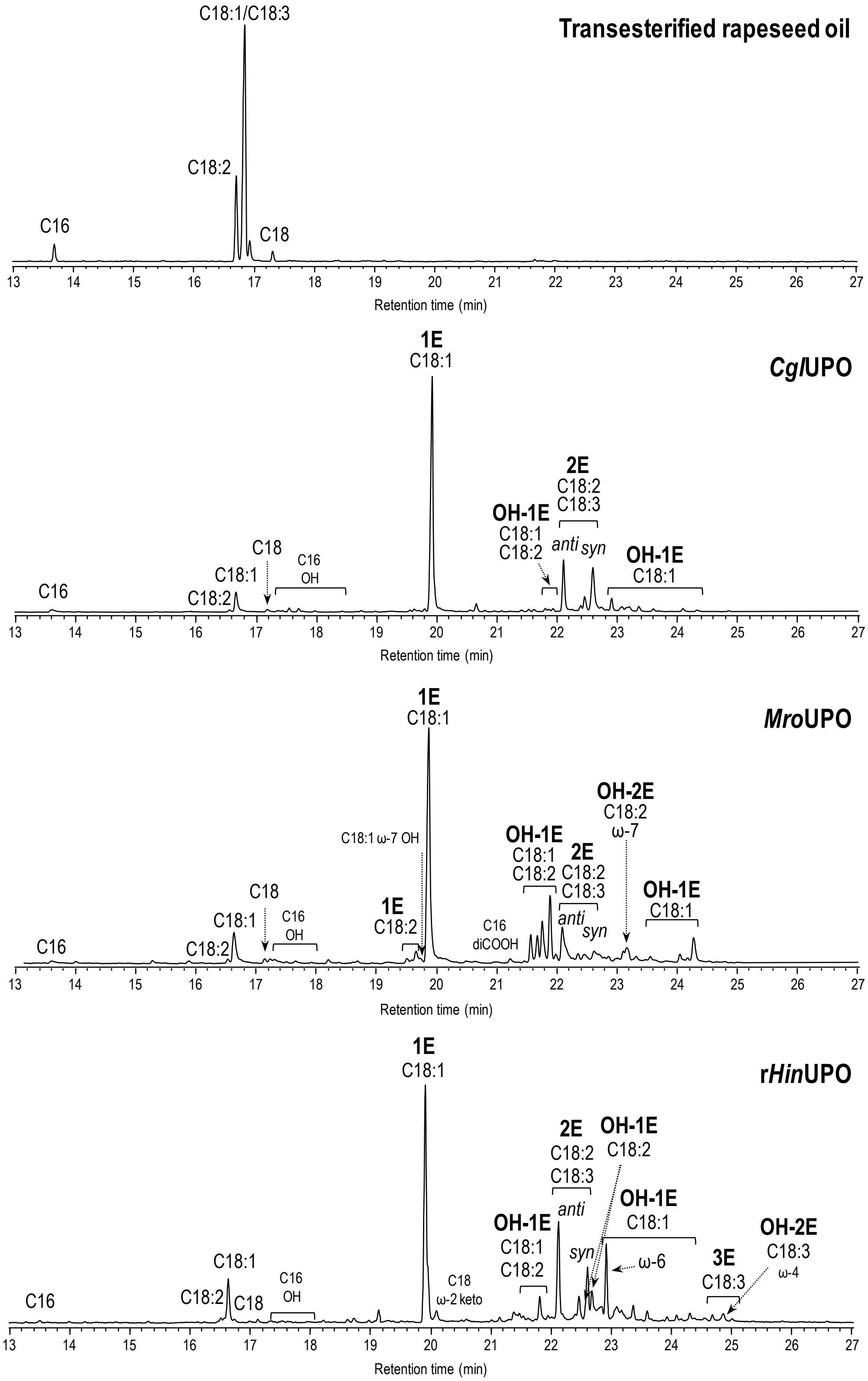

Supplementary Figure S10. GC-MS analysis of transesterified rapeseed oil (0.1 mM total fatty-acid concentration) reactions with *Cgl*UPO, *Mro*UPO and r*Hin*UPO (0.5 µM), showing monoepoxides, diepoxides (including *syn* and *anti* isomers from linoleic acid) and triepoxides (1E, 2E and 3E, respectively) and other oxygenated (hydroxy, OH; and keto) derivatives (often combined in the same compound). Control chromatogram is also shown in the top.

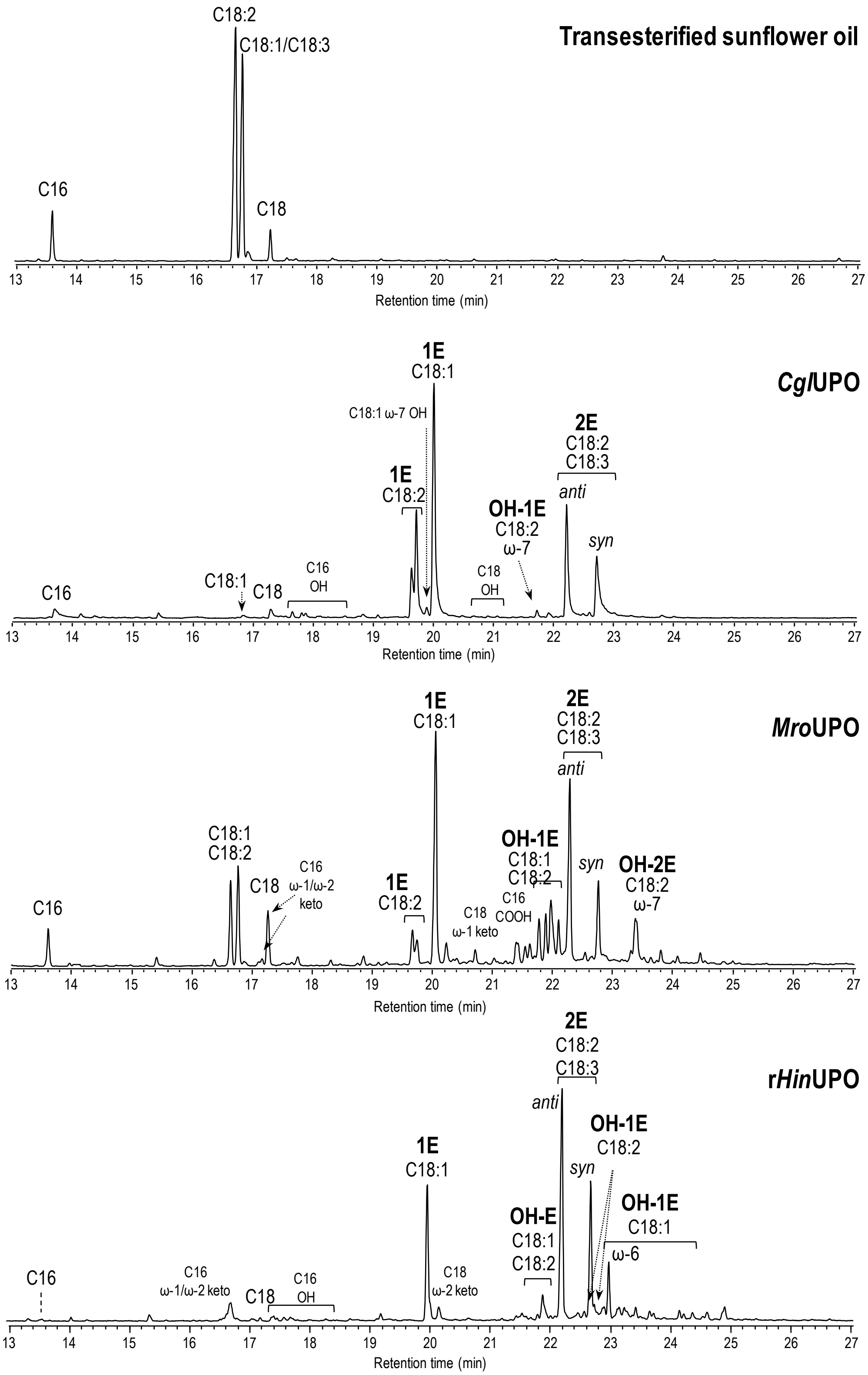

Supplementary Figure S11. GC-MS analysis of reactions of transesterified sunflower oil with *Cgl*UPO, *Mro*UPO and r*Hin*UPO (0.5 µM), showing monoepoxides and diepoxides (including *syn* and *anti* isomers from linoleic acid; 1E and 2E, respectively) and other oxygenated (hydroxy, OH; keto; and carboxy, COOH) derivatives of methyl esters. Control chromatogram is also shown in the top.

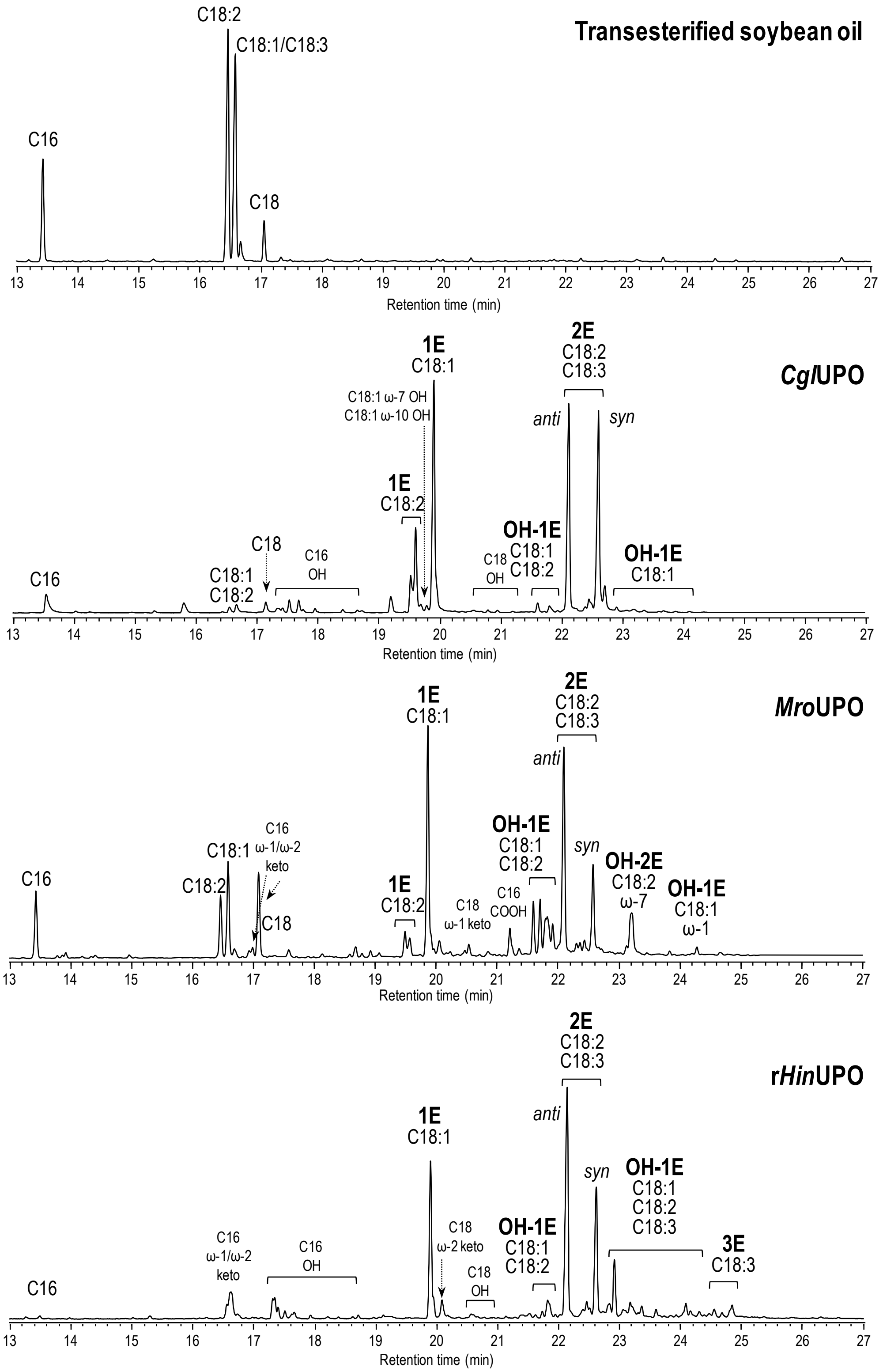

Supplementary Figure S12. GC-MS analysis of reactions of transesterified soybean oil with *Cgl*UPO, *Mro*UPO and r*Hin*UPO (0.5 µM), showing monoepoxides, diepoxides (including *syn* and *anti* isomers from linoleic acid) and triepoxides (1E, 2E and 3E, respectively) and other oxygenated (hydroxy, OH; keto; and carboxy, COOH) derivatives of methyl esters. Control chromatogram is also shown in the top.

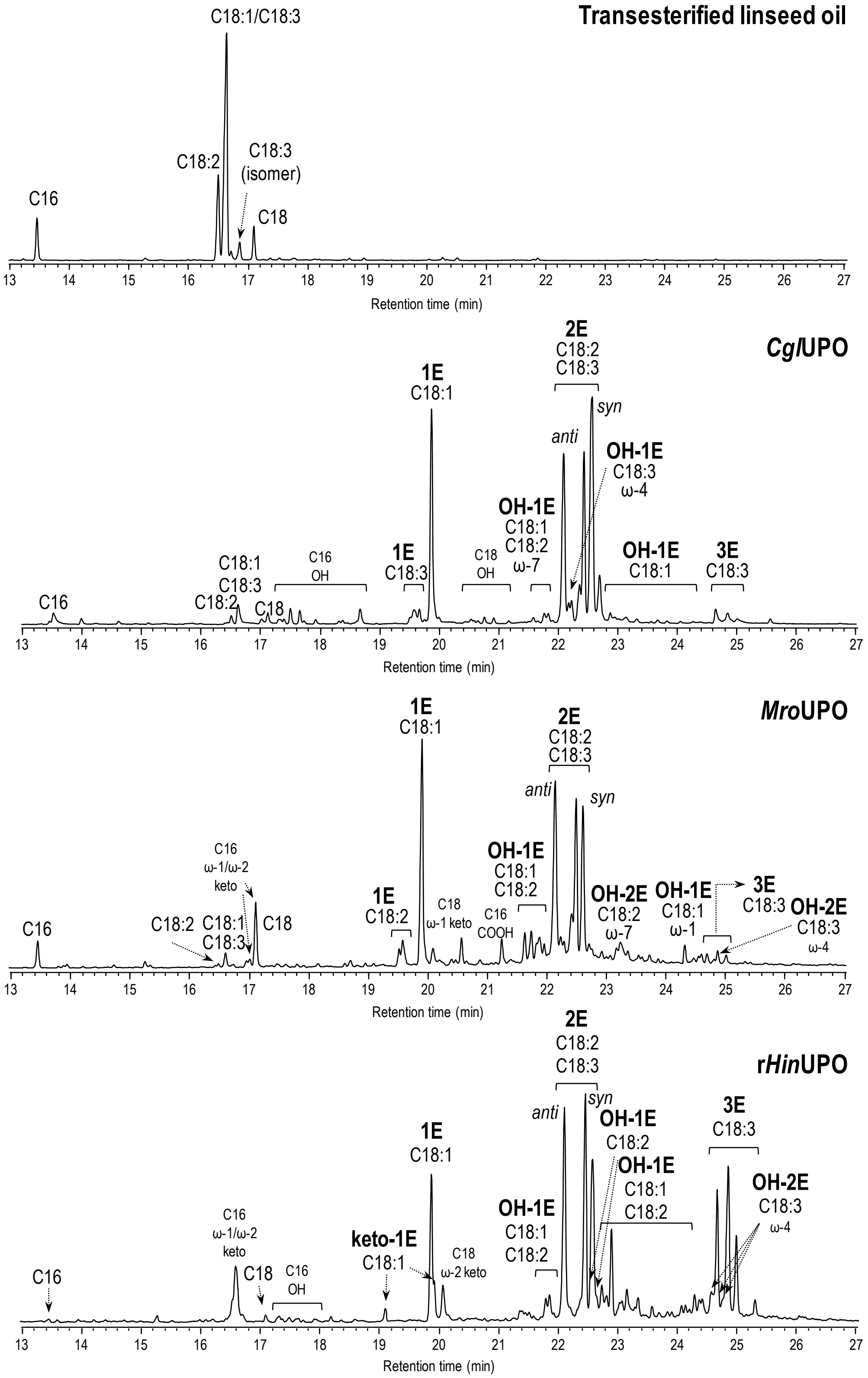

Supplementary Figure S13. GC-MS analysis of reactions of transesterified linseed oil with *Cgl*UPO, *Mro*UPO and r*Hin*UPO (0.5 µM), showing monoepoxides, diepoxides (including *syn* and *anti* isomers from linoleic acid) and triepoxides (1E, 2E and 3E, respectively) and other oxygenated (hydroxy, OH; keto; and carboxy, COOH) derivatives of methyl esters. Control chromatogram is also shown in the top.

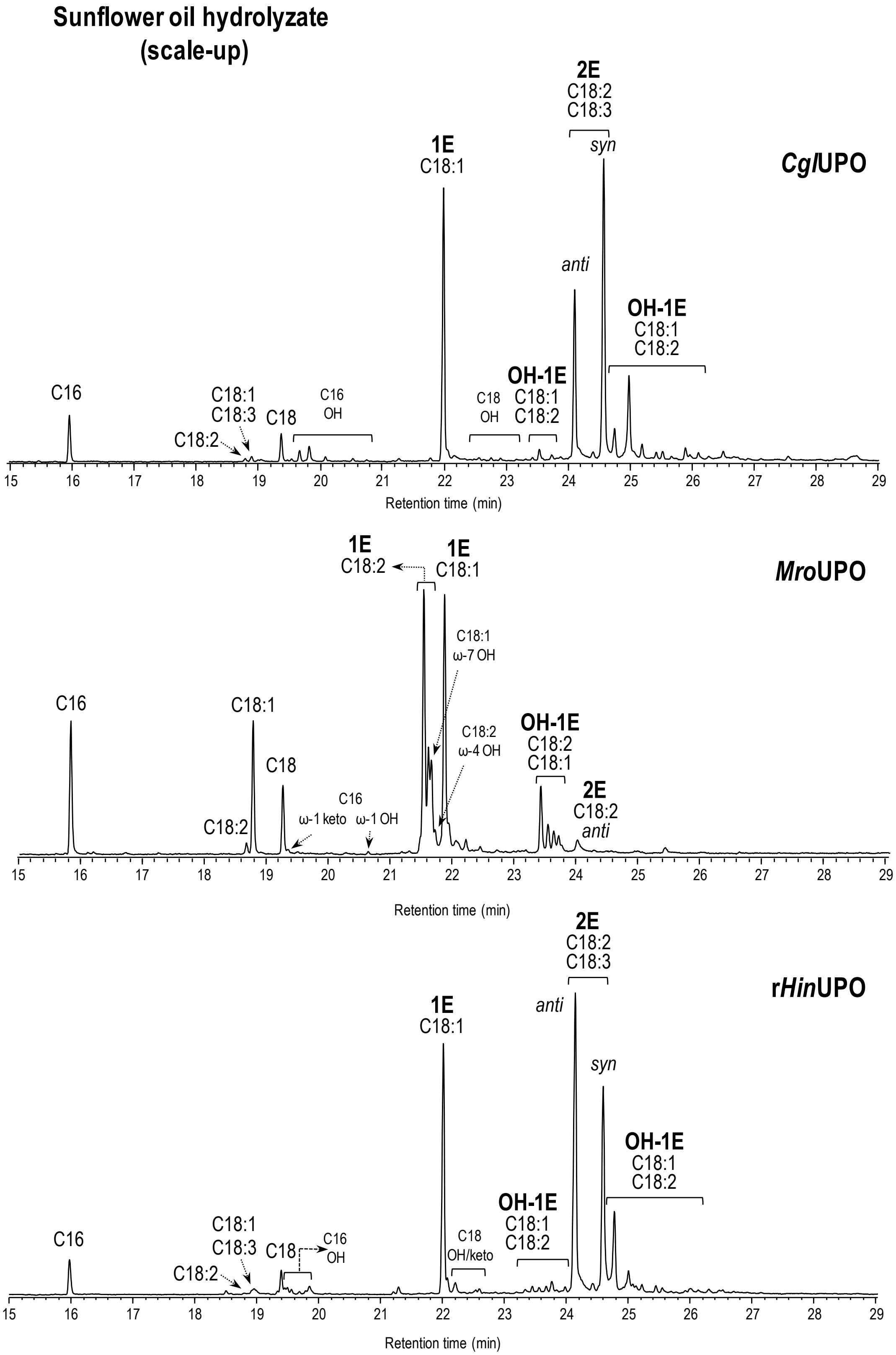

Supplementary Figure S14. GC-MS analysis of upscaled reactions of sunflower-oil hydrolyzate (30 mM total fatty-acid concentration) with *Cgl*UPO, *Mro*UPO and r*Hin*UPO (30 µM), showing monoepoxides and diepoxides (including *syn* and *anti* isomers from linoleic acid; 1E and 2E, respectively) and other oxygenated (hydroxy, OH; and keto) derivatives.
